# Post COVID-19 Organizing Pneumonia: The Right Time to Interfere

**DOI:** 10.3390/medicina57030283

**Published:** 2021-03-18

**Authors:** Kristina Bieksiene, Jurgita Zaveckiene, Kestutis Malakauskas, Neringa Vaguliene, Marius Zemaitis, Skaidrius Miliauskas

**Affiliations:** 1 Department of Pulmonology, Lithuanian University of Health Sciences, 44307 Kaunas, Lithuania; kestutis.malakauskas@lsmuni.lt (K.M.); neringa.vaguliene@lsmu.lt (N.V.); marius.zemaitis@lsmu.lt (M.Z.); skaidrius.miliauskas@lsmu.lt (S.M.); 2 Department of Radiology, Lithuanian University of Health Sciences, 44307 Kaunas, Lithuania; jurgita.zaveckiene@lsmu.lt

**Keywords:** COVID-19, viral pneumonia, organizing pneumonia, systemic glucocorticoids, interstitial lung diseases

## Abstract

The COVID-19 pandemic dramatically changed medical care. Healthcare professionals are faced with new issues. Patients who survived COVID-19 have plenty of different continuing symptoms, of which the most common are fatigue and breathlessness. It is not well known how to care for patients with persistent or worsening respiratory symptoms and changes on chest X-ray following COVID-19 pneumonia. In this article, we talk about a subgroup of patients with organizing pneumonia following COVID-19 pneumonia that could be effectively treated with systemic glucocorticoids. It is important that patients with COVID-19 pneumonia be followed-up at least three weeks after diagnosis, in order to recognize early lung damage. We are providing a management algorithm for early diagnosis of lung diseases after COVID-19 pneumonia.

The World Health Organization (WHO) had declared the Coronavirus disease 2019 (COVID-19) a pandemic by March 2020. For more than a year, human beings have faced the largest medical challenge. Globally, by the 4 March 2021, there have been 114,853,685 confirmed cases of COVID-19 and 2,554,694 deaths reported by the WHO [1]. There is a hope that the ongoing global vaccination will stop the pandemic in the near future.

The identified pathogen was a novel enveloped RNA beta coronavirus that has now been named severe acute respiratory syndrome coronavirus 2 (SARS-CoV-2) [2]. Most patients with COVID-19 have signs of respiratory infection. Some infected people experience a mild respiratory illness, and some others are completely asymptomatic. A part of patient’s progress to severe disease, with acute lung injury come multiple organ dysfunction and high mortality [3]. The critical illness that is characterized primarily by acute respiratory distress syndrome (ARDS) develops in 15% of hospitalized patients with COVID-19 [4,5,6,7]. Usually, they are older patients and/or have other poor prognostic factors included the presence of comorbidities (obesity, diabetes, hypertension etc.) and a history of smoking [5].

Viral pneumonia is the most common indication for hospital admission. More than 80% of patients are hospitalized to general medical wards and only a smaller proportion of patients are admitted to intensive care units (ICU) [8]. A Chinese study showed that from 1099 overall hospitalized COVID-19 patients, 16% had severe pneumonia and 5% required ICU admission [6]. COVID-19-related ICU mortality was reported to be from 16% to 78% [7,8,9,10,11,12,13,14,15,16].

A total of 200,884 cases of COVID-19 and 3294 deaths due to COVID-19 were reported by the Lithuanian Department of Statistics from March 2020. During the first wave of the pandemic the incidence of COVID-19 and the mortality rate in Lithuania were very low. Only 863 patients with COVID-19 were hospitalized and 102 of them died. However, during the second wave, Lithuania was among the leaders in Europe, in terms of the number of new COVID-19 cases. The rate of hospitalized patients reached 11,673 cases. [15].

Current COVID-19 guidelines mostly cover management of the acute disease, prevention, and vaccination. Less is known how to deal with the persistent symptoms after COVID-19. During the acute COVID-19 period, the extent of lung damage is proportional to the disease severity. However, it is still not completely known what predicts the severe course of COVID-19. Moreover, the severity of acute COVID-19 is not thought to be linked to the likelihood of development of persistent symptoms after the acute COVID-19 period [16]. People with positive a COVID-19 test could feel symptoms for more than 5 weeks. Symptoms are highly ranging and variable. The most common symptoms are breathlessness and fatigue [17]. 

There was no clear definition of “long COVID” until now. Recommendations basically are made on experts’ opinion. United guidelines of three organizations “COVID-19 rapid guideline: managing the long-term effects of COVID-19“were recently published in order to help clinicians [18]. Authors defined three forms of COVID-19 course: acute (signs and symptoms for up to 4 weeks), ongoing symptomatic (signs and symptoms of COVID-19 present from 4 weeks and up to 12 weeks) and post-COVID-19 syndrome (signs and symptoms that develop during or after an infection consistent with COVID-19, present for more than 12 weeks and are not attributable to alternative diagnoses). The authors underline, that it is very important that patients be informed about the possible course of COVID-19 and what steps should be taken if they have new, ongoing or worsening symptoms, especially if they have them for more than four weeks after the beginning of acute COVID-19 [18]. Pulmonologists, family physicians and cardiologists will have to deal with the plenty of such cases soon. 

We are working at the tertiary university hospital (Hospital of Lithuanian University of Health Sciences Kaunas Clinics). A total of 443 patients with COVID-19 were treated in our university hospital from March until December 2020. Viral pneumonia was diagnosed in 41.7% (*n* = 185) of these patients. Most of them—131 (70%) were treated in the ICU. The mortality, associated with SARS CoV2 pneumonia, for patients requiring ICU care was 56% [15]. The most severe COVID-19 patients at least three weeks after diagnosis of COVID-19 pneumonia were treated in our department of Pulmonology from December 2020. 

The aim of this article is to discuss the particular part of post COVID-19—patients with ongoing respiratory symptoms. Usually there are two groups of survived “unsuccessful” cases showing the signs of severe lung disease: (1) significant radiological changes on chest radiograph or computed tomography (CT); or (2) the demand of continuous invasive or non-invasive lung ventilation, high flow oxygen therapy or long-term oxygen therapy. A significant part of the latter group of patients develop chronic respiratory failure. We believe that previous lung disease and condition, concomitant diseases and their treatment are important [19]. The main question is whether and how we can help at this point in time. Recently, several papers have evaluated radiological patterns of different forms of diffuse parenchymal lung disease during the late course of COVID-19 [20]. COVID-19 pneumonia manifests on chest CT imaging with rapid evolution from focal unilateral to diffuse bilateral ground-glass opacities that progressed to or co-existed with consolidations within 1–3 weeks. Such abnormalities could be seen even in asymptomatic patients [20]. Various other forms of interstitial lung disease have also been proposed where more prominent fibrotic changes due to severe COVID-19 were evident [21]. Furthermore, recently the first report of single-centre nature on organizing pneumonia with longitudinal follow-up secondary to COVID-19-associated ARDS has been published [22]. Organizing pneumonia is generally sensitive to glucocorticosteroids. As we know, glucocorticosteroids (6 mg dexamethasone or equivalent given once daily for up to 10 days) are now recommended by WHO in severe and critical forms of COVID-19 [23,24]. It could decrease the incidence of organizing pneumonia on ARDS secondary to COVID-19 [25]. In general, we are looking for the patients with recent COVID-19 lung damage (“early post-acute COVID-19 phase”) and predominant signs of organizing pneumonia whom we can help with systemic glucocorticosteroids. As it was shown in a German study, the incidence of organizing pneumonia was 12.5% in a severe COVID-19 survivor’s cohort [22]. A Chinese study reported that, 3 weeks after symptom onset and disease progression, CT scans showed consolidation and ground glass opacities, bronchiolectasis, pleural effusion, thickening of the adjacent pleura, and (Figure 1 and Figure 2) [20]. Although the definitive diagnosis of organizing pneumonia requires histological assessment, biopsy usually cannot be performed due to the severe condition of patient [25,26]. If another interstitial lung disease is suspected, additional evaluation and diagnostic procedures might be needed (transbronchial biopsy etc.). There is still no evidence and it is under discussion, that current and emerging antifibrotic drugs could have therapeutic potential for treating the COVID-19 long-term fibrotic consequences that might follow this pandemic [21]. It is not clearly known if anti-inflammatory or antifibrotic medication will have any value in this pathology. The follow up CT shows the improvement or progression of lung lesions, reflecting therapeutic effects [20]. It is important that the survivors of severe and also moderate and mild COVID-19 should be followed-up regularly to recognize early lung damage [22]. A single centre Chinese study showed that two weeks after discharge might be the optimal time point for early radiological estimation for most of the patients [27]. It is believed, that the two weeks time point is the best time for patients with ongoing respiratory symptoms and changes on chest X-ray for follow up.

Patients with COVID-19 have increased risk of thromboembolic complications. The risk of venous thromboembolism (VTE) is higher in those with severe COVID-19, even when prophylactic anticoagulation is used [28]. The SARS-CoV-2 infection is associated with a number of coagulation abnormalities that include increased levels of d-dimer and fibrinogen, normal or slightly prolonged activated partial thromboplastin time, modestly prolonged prothrombin time, slightly decreased platelet counts and markedly hypercoagulable thromboelastometry profiles [29,30]. This COVID-19-associated coagulopathy increases the risk of pulmonary embolism [31]. A case reported study showed that acute pulmonary embolism could also complicate mild COVID-19 cases and it occurs late in the course of the disease [32].

Finally, we present our management algorithm for patients with early diagnosis of lung damage after COVID-19 pneumonia (Figure 3). This algorithm covers the patients with clinicoradiological confirmed COVID-19 pneumonia, after at least three weeks from diagnosis. Part of these patients continue treatment in hospital, others are discharged from hospital and the rest of them with COVID-19 pneumonia are treated as outpatients.

Patients still requiring inpatient care, who are suspected to have COVID-19 lung damage (having persistent respiratory symptoms, supplementary oxygen need, abnormal two view chest X–ray repeatedly), should be referred to a pulmonologist for assessment.

Patients with COVID-19 pneumonia, who have been discharged from the hospital or were treated as outpatients, should consult their family doctor in the coming in 2–4-week period. Family doctors should undertake clinical assessment and repeat two-views chest X-ray, blood count, C reactive protein, oxygen saturation measurements, electrocardiography. If the chest X-ray changes have not resolved and/or the patient’s respiratory symptoms are ongoing, COVID-19 lung damage is suspected, and hence referral to a pulmonologist should be considered.

Patients who are referred to the pulmonologist should proceed to a high-resolution CT scan and CT pulmonary angiogram to assess for the presence of possible interstitial lung disease (ILD) and pulmonary embolism (PE). Patients diagnosed with PE should be treated according to standard guidelines. If there is evidence of ILD, patients should undergo pulmonary function testing (spirometry, lung diffusion capacity), the 6 minutes’ walk test and lung biopsy according to the clinical situation. Finally, the patients are discussed in the ILD multidisciplinary team meeting together with the pulmonologist, radiologist and pathologist if their diagnosis and treatment of ILD is confirmed.

If there is no evidence of ILD or PE, another diagnosis should be considered and managed appropriately.

Mild COVID-19 patients (without COVID-19 pneumonia) are not included in this algorithm. Follow-up is not recommended if these patients do not have any respiratory symptoms. If patients’ respiratory symptoms continue or worsen they should consult their family doctor. Patients with ongoing radiographic changes but improving clinical symptoms have to follow this algorithm, because radiological changes which were not resolved could be related with lung cancer. The multidisciplinary rehabilitation team should work with these patients.

In conclusion, COVID-19 is a novel infection, being the largest medical challenge for more than a year. Lung damage, presenting with different interstitial changes, seen on a CT scan, could follow COVID-19 pneumonia. One of them—organizing pneumonia—is regarded as glucocorticoids sensitive. Patients with post COVID-19 pneumonia have to be evaluated to recognize early lung damage. We are hoping that our proposed management algorithm of post COVID-19 pneumonia could be another step to improve the care of these patients and result in less consequential outcomes.

## Figures and Tables

**Figure 1:**
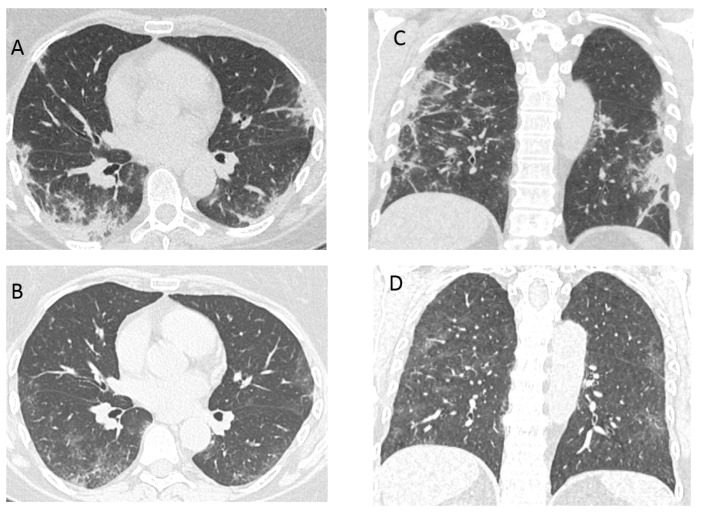
(**A**,**C**) Computed tomography (CT) of a 58-year-old woman, 21 days after COVID-19 pneumonia diagnosis, treated at home, with persistent and deteriorating respiratory symptoms. Peripheral consolidation with air bronchograms, reticular changes and small areas of ground glass opacities (organizing pneumonia). (**B**,**D**) CT after 4 weeks of treatment with systemic glucocorticoids—resorption of consolidation with the fibrotic component (reticular changes, bronchiectasis).

**Figure 2:**
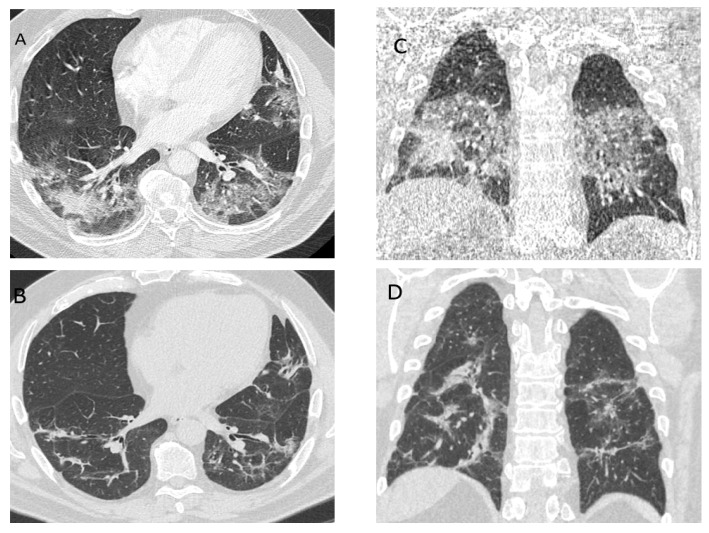
A 59-year-old man, with severe COVID-19 pneumonia treated in hospital. (**A**,**C**) At 10 days after the onset of symptoms. Peribronchovascular, perilobular consolidation and ground-glass opacities—organizing pneumonia and nonspecific interstitial pneumonia. (**B**,**D**) At 2 months after diagnosis with persistent respiratory symptoms; the patient was not treated with oral glucocorticoids. Decreased areas of ground glass opacities, small consolidation with air bronchograms, fibrotic changes.

**Figure 3:**
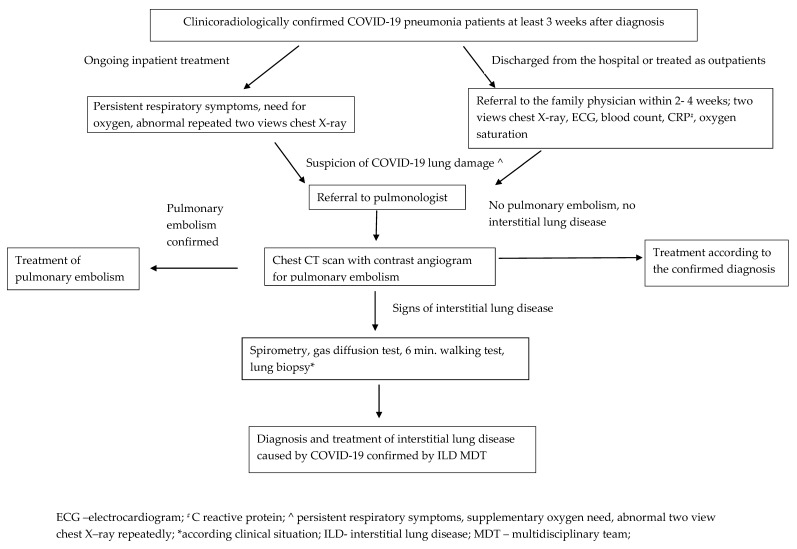
Management algorithm for early diagnosis of lung disease after COVID-19 pneumonia.

## Data Availability

Not applicable.
